# A novel mutation affecting the arginine‐137 residue of AVPR2 in dizygous twins leads to nephrogenic diabetes insipidus and attenuated urine exosome aquaporin‐2

**DOI:** 10.14814/phy2.12764

**Published:** 2016-04-25

**Authors:** Gitte R. Hinrichs, Louise H. Hansen, Maria R. Nielsen, Christina Fagerberg, Hans Dieperink, Søren Rittig, Boye L. Jensen

**Affiliations:** ^1^ Department of Cardiovascular and Renal ResearchInstitute of Molecular MedicineUniversity of Southern DenmarkOdenseDenmark; ^2^ Anaesthesiology and Intensive CareOdense University HospitalOdenseDenmark; ^3^ Clinical GeneticsOdense University HospitalOdenseDenmark; ^4^ Department of NephrologyOdense University HospitalOdenseDenmark; ^5^ Department of PaediatricsAarhus University HospitalAarhusDenmark

**Keywords:** ALIX, dehydration, receptor, vasopressin

## Abstract

Mutations in the vasopressin V2 receptor gene *AVPR2* may cause X‐linked nephrogenic diabetes insipidus by defective apical insertion of aquaporin‐2 in the renal collecting duct principal cell. Substitution mutations with exchange of arginine at codon 137 can cause nephrogenic syndrome of inappropriate antidiuresis or congenital X‐linked nephrogenic diabetes insipidus. We present a novel mutation in codon 137 within *AVPR2* with substitution of glycine for arginine in male dizygotic twins. Nephrogenic diabetes insipidus was demonstrated by water deprivation test and resistance to vasopressin administration. While a similar urine exosome release rate was shown between probands and controls by western blotting for the marker ALIX, there was a selective decrease in exosome aquaporin‐2 versus aquaporin‐1 protein in probands compared to controls.

## Introduction

Water balance is achieved through thirst‐stimulated water intake and regulated water excretion by urine. Renal water handling is governed by osmolality‐regulated release of the hormone arginine vasopressin (AVP) from the posterior pituitary gland. AVP stimulates urine concentration by increasing water permeability of renal collecting ducts through activation of the vasopressin‐2 receptor (V2R) in principal cells. Binding of the ligand initiates a cascade of events by which cyclic AMP through PKA phosphorylates aquaporin‐2 (AQP2) at multiple sites and promotes insertion into the apical membrane of the cells thereby facilitating water permeability and concentration of urine. Clinical disorders of defective urinary concentration include central diabetes insipidus with AVP deficiency and nephrogenic diabetes insipidus (NDI) caused by inability of the kidneys to respond appropriately to AVP. Primary forms of NDI may be caused by mutations of the V2R gene, *AVPR2*, located on the X‐chromosome. More than 200 mutations have been described comprising deletions, insertions, nonsense, and missense rearrangements. In the protein sequence, the arginine residue at position 137 is of major significance since mutations may cause constitutive activation of V2R resulting in nephrogenic syndrome of inappropriate antidiuresis (N‐SIAD) or X‐linked nephrogenic diabetes insipidus (NDI) due to loss of function of the V2R (Feldman et al. 2005; Morin et al. 2012; Fu et al. 2013). The purpose of this report is to characterize the phenotype of a family with a novel mutation not previously described and to test the hypothesis that urine exosomes could serve as a noninvasive diagnostic tool to reveal altered apical membrane transport protein abundance, notably AQP2.

## Case Report

**Figure 1:**
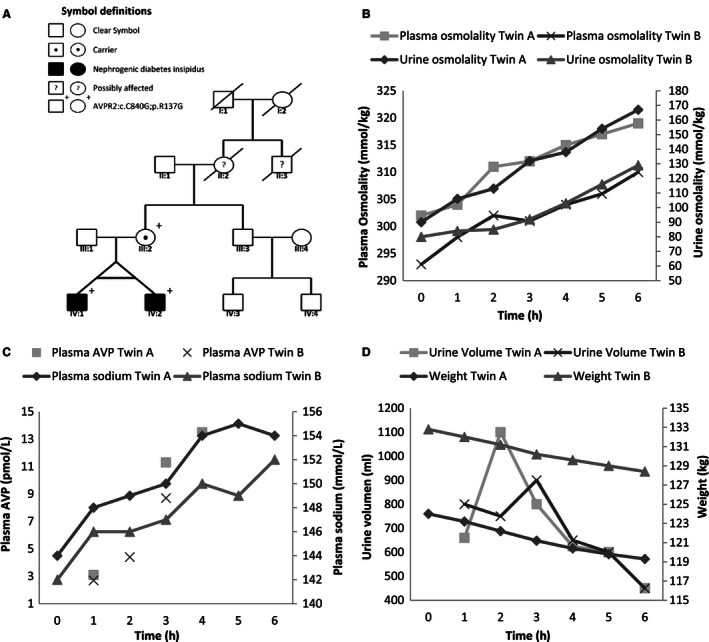
(A) Pedigree. (B–D) Diagrams show the effect of water deprivation test. (B) Increasing plasma and urine osmolality in response to water deprivation. (C) Changes in plasma AVP concentrations and plasma sodium concentrations during water deprivation. Both increased significantly. (D) Diagram shows diuresis and weight; probands displayed continuous high diuresis despite water deprivation and weight loss.

The case concerns dizygotic male Caucasian twins, age 24 years at the time of reporting. Their symptoms were first noted at 8 months of age with delayed mental and motor skills and failure to thrive. Abnormal fluid intake and diuresis were noted along with hypernatremia, serum hyperosmolality, low urinary osmolality, and hyponatriuria. During childhood, water deprivation test was attempted and arginine vasopressin was administered, but had unremarkable effects on diuresis and urine osmolality. At the same time, the mother revealed mild symptoms of excessive fluid intake and diuresis and the patients were diagnosed clinically with presumably X‐linked NDI. The patients were treated with thiazide diuretics and unlimited amounts of fluid, after which they thrived. At age 21 years, the patients stopped all medication due to adverse effect. Three years later the patients were seen at nephrology ward and it was decided to perform a standard diagnostic test for urine concentration ability and perform a genetic analysis. Pedigree is shown in Figure 1A.

## Methods

The examination ran as standard diagnostic procedure at the Department of Nephrology, Odense University Hospital. No extra samples or procedures were introduced with other purpose than diagnosis. It was approved as a diagnostic procedure by The Regional Scientific Ethics Committee for Southern Denmark.

### Water deprivation test

Baseline laboratory blood and urine samples were obtained (Table 1), and water deprivation test followed by the administration of AVP was carried out under constant medical supervision. Free fluid intake was allowed until 1 h before the test began. If the sodium level rose above 150 mmol/L or weight loss increased above 5% of their weight to time = 0, desmopressin was administered. During the test, their well‐being was monitored continuously and blood pressure, pulse, weight, and urinary output were measured hourly and plasma sodium analyzed immediately after sampling. Plasma AVP concentration was measured three times during the test.

**Table 1:** Clinical characteristics of the probands

Parameter	Patient A	Patient B	Age‐matched control
Weight (kg)	124	132.8	
Blood pressure (mmHg)			
Systolic	133	124	
Diastolic	85	94	
Heart rate, min	88	98	
Serum and plasma studies			
Erythrocytes vol. Fr.	0.5	0.48	0.40–0.50
Hemoglobin (g/L)	10.3	10.2	8.3–10.5
Platelets (10^9^/L)	234	220	145–350
Leukocytes (10^9^/L)	7.89	7.8	3.50–8.80
Albumin (g/L)	44	43	36–50
Calcium ion (mmol/L)	1.29	1.25	1.18–1.32
Phosphate (mmol/L)	0.62	0.88	0.71–1.53
Creatinine (*μ*mol/L)	70	76	60–105
Carbamid	3.8	3.8	3.2–8.1
Sodium (mmol/L)	144	142	137–145
Potassium (mmol/L)	3.4	3.1	3.5–4.4
Thyrotropin (10^−3^ IU/L)	1.7	3.7	0.30–4.0
Thyroxine (nmol/L)	80	82	60–130
Triiodthyronin (nmol/L)	2.1	2.1	1.3–2.2
Cortisol (nmol/L)	488	635	200–700
Glucose (mmol/L)	5.1	6	–
Alanine transaminase (U/L)	14	19	10–70
Bilirubin (*μ*mol/L)	14	15	5–25
Creatinine kinase (U/L)	32	36	50–400
Lactate dehydrogenase (U/L)	213	187	105–205
Urine studies			
Albumin, U (mg/L)	<5	<5	<20
Osmolalitet, U (mmol/kg)	90	80	50–1400
Sodium, U (mmol/L)	25	<20	–
Ultrasound, kidney	Normal	Hydronephrosis	

### Exosome analysis

Urinary exosomes were isolated from three spot urine samples (100 mL) from each patient. The urine samples were collected at time 0 h (before water deprivation), 4 h into the test, and after vasopressin administration. All urine samples were immediately supplemented with two protease inhibitor complete tablets (Roche, Mannheim, Germany) and stored at −80°C until recovery of exosomes. The urine samples thawed over night at 4°C and were then thoroughly vortexed and subsequently centrifuged at 1363 *g* for 30 min at 4°C. Exosomes were pelleted by ultracentrifugation of the supernatant at 220,000 *g* for 100 min at 4°C. The pellet was resuspended in 2 × 100 *μ*L resuspension buffer (sucrose: 0.3 mol/L; imidazole: 25 mmol/L; EDTA–disodium salt: 1 mmol/L; pH 7.2; complete mini tablet; Roche). The amount of resuspended pellet from ultracentrifugation used for each western blot was calculated based on creatinine concentration in the original urine samples (Zachar et al. 2015). To each sample 10X reducing agent (NuPAGE, Biorad, Hercules, CA) and 4x LDS sample buffer (NuPAGE, Biorad) were added. The samples were denatured for 5 min at 95°C. The samples were loaded on a 4–15% tris‐HCl SDS‐PAGE gel (Biorad) or a 4–12% Bis‐Tris, 10 wells (Invitrogen, Carlsbad, CA). Separation ran with a constant voltage of 200 V for 40–60 min using 1x TGS (10x TGS, Tris‐glycine‐SDS, Biorad) as running buffer or 1x Mes‐buffer (NuPAGE 20x MES SDS running buffer, Invitrogen). Precision Plus Protein Standard (All Blue, Biorad) was used as a size marker. The samples were transferred to a PVDF membrane (0.45 *μ*m pore size, Immobilon P; Millipore, Billerica, MA) at voltage of 35 V for 60 min. 1X Transfer buffer (Nupage20X transfer buffer in 10% ethanol) was used as running buffer. Prior to blotting, the membrane was activated with 99.9% ethanol for 30 sec, then washed in Milli Q water for 1 min, and afterward placed in transfer buffer. The membrane was blocked in 5% skim milk/TBST over night at 4°C or for 1 h at room temperature at a shaking table. The membrane was washed 3 × 10 min in TBST. For visualization, ECL plus Western Blotting Detection System (Amersham Bioscience/GE Healthcare Buckinghamshire, UK) was applied to the membrane for 1 min. An image of the membrane was made by exposure to X‐ray film. The primary antibodies used were goat anti‐AQP2 (1:2000 in 5% skim milk in TBST buffer; sc‐9882, Santa Cruz Biotechnology c17, Lot. no. K1711, Santa Cruz Biotechnology, Dallas, TX), rabbit anti‐AQP1 (1:3000; ab‐15080, Abcam Cambridge, UK), mouse anti‐ALIX (1:100 in 5% skim milk in TBST buffer; 3A9, Monoclonal IgG, Santa Cruz Biotechnology #sc‐53538). Primary antibodies were detected with HRP‐conjugated secondary antibody (Dako, Glostrup, Denmark) and developed using ECL mixture (Amersham, Birkerod, Denmark).

## Results

**Figure 2:**
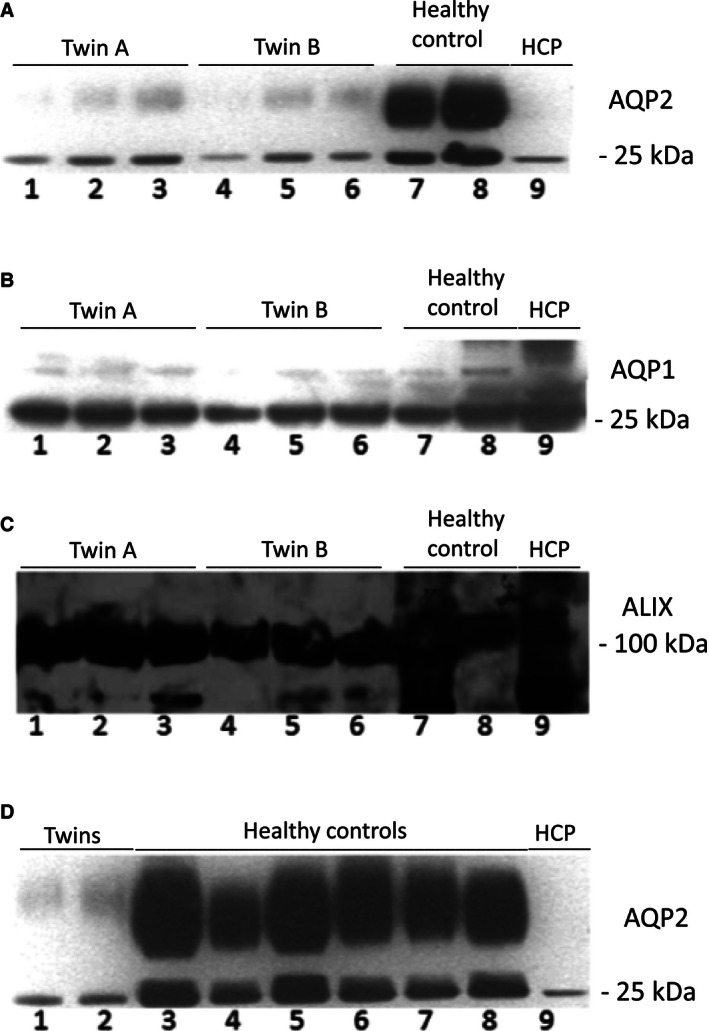
Results of western immunoblotting experiments with urine exosomes from probands and controls. A similar amount of creatinine was loaded into each well and thus a larger absolute volume of urine for the patients compared to controls. In A–C, each lane on the SDS‐PAGE contained: 1, time 0 h; 2, 4 h of WD test; 3, 2 h after AVP administration; 4, time 0 h; 5, 4 h of WD test; 6, 2 h after AVP administration; 7, healthy control 1; 8, healthy control 2; 9, human kidney cortex pool. (A) Aquaporin‐2. Predicted molecular weight: 25 kDa and glycosylated at 36 kDa. Abundance increased slightly with water deprivation in probands. Abundance was markedly lower in patients compared to two healthy controls. (B) Immunoblotting of urine exosome fraction for aquaporin‐1. Predicted molecular weight: 32 kDa. Numbering was as above. There was a similar level of AQP1 in exosomes across conditions and in patients and controls. (C) Immunoblotting of urine exosome fraction for an exosome‐specific protein (“ALIX”). A prominent protein that migrated in the range ~100 kDa was detected in both probands and controls and in human kidney homogenate (predicted size 95 kDa). (D) Western immunoblotting of urine exosome fraction for aquaporin‐2. Exosomes from last collection period in patients were compared to six healthy controls in habitual water status. Volume of urine loaded was normalized for creatinine content. Patients excreted less AQP2 even after severe dehydration compared to water‐replete persons. The lanes on the SDS‐PAGE contained: 1, 4 h of WD test twin A; 2, 4 h of WD test twin B; 3–8, healthy controls; 9, human kidney cortex pool.

Ultrasound examination of the kidneys revealed normal conditions in sibling A, but sibling B had bilateral hydronephrosis. Sequencing of the *AVPR2* gene with DNA from leukocytes revealed a hemizygous mutation in exon 2 of *AVPR2* in both patients: *AVPR2:c.C840G;p.R137G*. The mother was heterozygous for this mutation (Fig. 1A). Water deprivation resulted in an increase in plasma [Na^+^] that prompted administration of AVP after 4.5 h. For both patients, plasma creatinine, carbamide, and potassium concentrations did not change and were within normal range. Water deprivation resulted in 10 mmol/L increase in plasma [Na^+^] (Fig. 1C), a ~17 mosmol/L increase in plasma osmolality (Fig. 1B), weight loss of 4 kg (3% of body weight), cumulated urine output ~4.3 and ~4.1 L (Fig. 1D), an increase in urine osmolality by 50% from low level (100 mosmol/L) at baseline, and a four times increase in plasma [AVP] (Fig. 1C). Sibling A had stable blood pressure (133/85 mmHg) and heart rate (mean 88/min) during the test. Sibling B showed variable blood pressure range 98/80 to 140/100 mmHg, and heart rate 72–122 bpm. By immunoblotting, AQP2 was detectable in urine exosomes from patients and normohydrated control persons (Fig. 2A). Despite loading similar amount of creatinine in a much larger volume of urine from patients onto the gel before SDS‐PAGE separation, AQP2 abundance was lower for the water‐deprived patients compared to the controls throughout (Fig. 2A). AQP2 protein abundance increased in both patients with water deprivation (Fig. 2A). By contrast, aquaporin‐1 (AQP1) abundance was not different between patients and control persons and did not change visibly with water deprivation (Fig. 2B). To verify that the signal derived from exosomes and obtain a measure for exosome release rate, western blotting for multivesicular body/exosome marker apoptosis‐linked gene‐2 interacting protein (ALIX) was performed. It revealed a single significant band with no marked difference in abundance with water deprivation and between controls and patients which indicate a roughly similar release rate of exosomes from the renal tubular epithelium (Fig. 2C). When comparing the patient samples obtained at the time with highest circulating AVP with the six healthy water‐replete controls, AQP2 abundance was significantly lower (Fig. 2C).

## Discussion

The present case presents dizygotic male twins with NDI characterized by repeated dehydration episodes in early infancy, polyuria, and polydipsia. We identified a not previously described X‐linked missense mutation in exon 2 of the *AVPR2* gene, C840G, which causes the amino acid shift R137G reflected by decreased AQP2 but not AQP1 in urine exosomes.

The water deprivation test confirmed the nephrogenic cause. There was significant, physiologically appropriate, increase in plasma AVP concentration, but with little effect on urine parameters. There was a continuous high urine output, low urine osmolality, and impaired ability to increase urine osmolality to normal levels. Addition of exogenous AVP analog corroborated this observation by having a marginal influence on urine osmolarity. The novel R137G amino acid substitution is located in the intracellular part of the receptor at the junction of the third transmembrane domain and second intracellular loop. The arginine residue at position 137 in the protein product of this gene is pivotal for normal function. Published mutations in this codon reveal opposite phenotypes. Feldman et al. (2005) described two infants with missense mutations in *AVPR2* exchanging arginine for leucine or cysteine. The clinical phenotype was inappropriate antidiuresis with undetectable AVP in plasma and increased capacity for cAMP production in cells with heterologous expression of the mutated *AVPR2* compared to wild type. This indicated a constitutively active V2R in these infants. The condition was named “nephrogenic syndrome of inappropriate antidiuresis” (N‐SIAD). The activation mutations R137L and R137C conferred pathological water retention and hyponatriemia. In contrast, the R137H mutation (Barak et al. 2001) was associated with an NDI phenotype similar to the present R137G mutation. The R137H mutation led to a phosphorylated receptor protein that was sequestered in intracellular vesicles, even in the absence of agonist, and therefore an impaired cAMP responsiveness to AVP. Whether a similar change is associated with the R137G mutation presented here was not resolved by the present study (Barak et al. 2001). Impaired cAMP responsiveness in principal cells would affect AQP2 transcription, translation, stability, and trafficking (Christensen et al. 1998). Loss‐of function mutation in AVPR2 in patients were associated with lower AQP2 protein in urine as determined by immunblotting (Kotnik et al. 2007). Changes in apical membrane abundance of AQP2 are reflected in urine exosomes in vitro and in experimental animals in vivo (Street et al. 2011). Exosomes originate from all nephron segments (Pisitkun et al. 2004; Fang et al. 2013), and the present study showed that based on an exosome marker protein, ALIX, patients and controls displayed rather similar overall release rate of exosomes. The similar abundance in exosomes between probands and patients of the proximal tubule‐associated AQP1 protein is in accordance with identical membrane association and release rate despite differences in water status and AVP in plasma. By contrast, the patients excreted significantly less exosomal AQP2 protein despite maximal endogenous and exogenous AVP stimulation compared to water‐replete control persons. The measurement of urine exosome AQP abundance could provide a noninvasive readout for vasopressin‐controlled epithelial events that may aid phenotypic characterization of rare inherited transport protein disorders in human patients.

## Conflict of Interest

None declared.
